# Minimal Stimulation *In Vitro* Fertilization: A Better Outcome 

**DOI:** 10.22074/ijfs.2016.4919

**Published:** 2016-06-01

**Authors:** Adrija Kumar Datta

**Affiliations:** Department of Reproductive Medicine, CREATE Fertility, Birmingham, UK

In *in vitro* fertilization (IVF) programme, the advantages of mild-stimulation have long been appreciated, while there was a called for more patient-friendly approach in ovarian stimulation around 20 years ago (1). However, the concept is yet to get wide-spread acceptance in the IVF community. The main impediment has been a lack of robust outcome data that can assure the success of mild-IVF at least as good as those of conventional IVF. The randomized controlled trials (RCTs) that compared sequential clomiphene citrate (CC) and low-dose gonadotropins (as mild/ minimal stimulation) with conventional long protocol were either small in sample size or heterogeneous in character (2). Nevertheless, recent meta-analyses and systematic reviews found no difference in pregnancy rates or live birth rates (LBRs) between sequential CC-gonadotropin protocol and conventional IVF (3, 4). More recently, a prospective cohort of 163 good prognosis patients undergoing IVF with sequential CC and low-dose gonadotropin regimen reported a cumulative-LBR (C-LBR) of 70% from a fresh and subsequent frozen embryo transfer (ET) up to 3 cycles (5). A large retrospective cohort study of 20, 244 cycles from Japan using a protocol comprising of extended CC (up to the trigger day)+gonadotropin and subsequent single vitrified-thawed ET found the treatment outcomes of in all age-groups were comparable with those in the Registry of the Society for Assisted Reproduction (SART) in the USA (6).

The article by Zhang et al. (7) intended to improve the treatment outcomes of minimal stimulation IVF by introducing certain modifications. They recommended the following protocol that was almost identical to the aforementioned Japanese study: extended course of CC up to the day of trigger, the final maturation of oocytes (trigger) by gonadotropin-releasing hormone (GnRH)-agonist and subsequent vitrified-thawed ET. In this protocol, human chorionic gonadotropins (hCG) as trigger was considered only if the couples insisted on fresh ET. The authors reminded us of the proven advantages of mild/ minimal stimulation protocol, especially with regards to its safety and patients’ tolerance. Each of the suggested modifications in the course of minimal-IVF cycles was backed up by recent evidence supporting improved clinical outcomes: GnRH-agonist trigger has been shown to increase oocytes maturation, while better LBR and perinatal outcome have now been linked with frozen-thawed ET. A distinct advantage of this regimen which the authors’ group had shown in a previous publication, was its better outcome in treating over-weight women (7). Not the least, continuation of CC throughout the follicular phase, by preventing the luteinizing hormone (LH) surge, effectively circumvented the need for expensive GnRH-antagonists. Due to its simplicity and savings on the cost of medications, the suggested minimal stimulation protocol could potentially be considered in low-resourced communities worldwide. As a whole, this strategy seems to be a step forward in establishing a successful patientfriendly IVF programme.

Even though the protocol sounded attractive theoretically, the crude data-evidence on the treatment success has still been limited to few retrospective studies and a yet unpublished RCT (n=564) by the authors’ own team. By randomly allocating good prognosis patients between mini-IVF with single ET and conventional IVF with double ET, the authors found lower cumulative C-LBRs (6 months) with the mini-IVF protocol [49 vs. 63%; relative risk (RR): 0.76; 95% confidence interval (CI) 0.64-0.89], albeit no incidence of ovarian hyperstimulation syndrome. The proposed strategies, therefore, need further evaluation through RCTs, by directly comparison of its LBRs per single ET (fresh or frozen), as well as C-LBRs with those of conventional IVF.

The publication by Zhang et al. (7) was not a review article in true sense. It was an effort to disseminate a certain minimal stimulation IVF-ET protocol with specific modifications on the ovulation trigger and ET strategies. In some places, the proposed treatment plan appeared rather too inflexible and specific. For example, it was not convincing why buserelin as a trigger had to be administered by intranasal route only (other than protecting patients from another needle-prick), or why frozen ET was recommended on a medicated cycle only, overlooking potential cost-savings on a natural cycle. Also, routine pre-treatment with combined contraceptive pill remained questionable. General acceptability of the recommended strategy might be restricted by the fact that not all embryology laboratories run an effective vitrification programme, and that the tariff of additional interventions e.g. freezing-thawing and storage of embryos for all patients may be considered as a limiting factor for many clinics. There was evidence from a number of RCTs that mild-IVF cycles, where fresh ETs were performed, resulted in a significant financial benefit, as compared to conventional IVF (4, 8, 9). However, comparative data on the cost-effectiveness of obligatory frozenthawed ET versus fresh ET in the setting of mild/ minimal-IVF are lacking.

The bulk of evidence of better LBRs and superior perinatal outcomes in frozen ET are largely derived from studies with conventional IVF (10). A compromised endometrial receptivity secondary to supra-physiological estrogen and progesterone levels following conventional ovarian stimulation has been implicated (11). Pre-trigger serum estrogen and progesterone levels that were lower than those of conventional IVF caused better endometrial receptivity following milder stimulation IVF and fresh ET (12). In fact, a meta-analysis found better implantation rates in mild-stimulation IVF (2). Adverse perinatal outcomes including low-birth weight and preterm birth have also been linked with the higher number of retrieved oocytes and high late follicular estrogen levels in conventional IVF, not with mild-IVF (10, 13). The mean birth-weight has been found to be higher following natural modified protocol than of conventional IVF (14). Until more evidence in support of using vitrified-thawed embryos in mild-IVF programme is available, the practice of fresh ET seems to continue. The compulsion of frozen ET in the protocol proposed by Zhang et al. (7) actually originated from the deleterious effects of both GnRH-agonist and CC (without gonadotropin) on endometrial receptivity. The former agent is known to be responsible for a luteal phase insufficiency, while the latter tends to cause endometrial thinning. Future studies may explore the possibility of fresh ET in this situation that is possible by replacing CC with tamoxifen (which does not affect endometrial thickness and has successfully been used in patients with estrogen-sensitive cancer) and by applying the emerging methods of enhancing luteal phase support following agonist trigger (15, 16). There was some evidence that sequential addition of CC in an antagonist cycle might improve the corpus luteal function by maintaining a good LH level in both follicular and luteal phase (17). Extrapolating this benefit of CC in GnRH-agonist-triggered cycles, a study found no rectification of the luteal defect induced by agonist trigger (18). Although the peak luteal LH and progesterone levels were elevated, the duration of luteal activity was no different from that of GnRH agonist-induced LH surge in this study. It would be interesting to examine if extended course of anti-estrogens up to the day of trigger, as proposed by Zhang et al. (7), could uphold the LH levels long enough to adequately support the luteal phase.

## References

[1] Edwards RG, Lobo R, Bouchard P (1996). Time to revolutionize ovarian stimulation. Hum Reprod.

[2] Verberg MF, Macklon NS, Nargund G, Frydman R, Devroey P, Broekmans FJ (2009). Mild ovarian stimulation for IVF. Hum Reprod Update.

[3] Gibreel A, Maheshwari A, Bhattacharya S (2012). Clomiphene citrate in combination with gonadotropins for controlled ovarian stimulation in women undergoing in vitro fertilization. Cochrane Database Syst Rev.

[4] Figueiredo JB, Nastri CO, Vieira AD, Martins WP (2013). Clomiphene combined with gonadotropins
and GnRH antagonist versus conventional controlled ovarian
hyperstimulation without clomiphene in women undergoing
assisted reproductive techniques: systematic review and meta-analysis. Arch Gynecol Obstet.

[5] Ferraretti AP, Gianaroli L, Magli MC, Devroey P (2015). Mild ovarian stimulation with clomiphene citrate launch is a realistic option for in vitro fertilization. Fertil Steril.

[6] Kato K, Takehara Y, Segawa T, Kawachiya S, Okuno T, Kobayashi T (2012). Minimal ovarian stimulation combined with elective single embryo transfer policy: age-specific results of a large, single-centre, Japanese cohort. Reprod Biol Endocrinol.

[7] Zhang JJ, Feret M, Chang L, Yang M, Merhi Z (2015). Obesity adversely impacts the number and maturity of oocytes in conventional IVF not in minimal stimulation IVF. Gynecol Endocrinol.

[8] Ragni G, Levi-Setti PE, Fadini R, Brigante C, Scarduelli C, Alagna F (2012). Clomiphene citrate versus high doses of gonadotropins for in vitro fertilisation in women with compromised ovarian reserve: a randomised controlled noninferiority trial. Reprod Biol Endocrinol.

[9] Heijnen EM, Eijkemans MJ, De Klerk C, Polinder S, Beckers NG, Klinkert ER (2007). A mild treatment strategy for invitro fertilisation: a randomised non-inferiority trial. Lancet.

[10] Pinborg A, Wennerholm UB, Romundstad LB, Loft A, Aittomaki K, Soderstrom-Anttila V (2013). Why do singletons conceived after assisted reproduction technology have adverse perinatal outcome?. Systematic review and metaanalysis. Hum Reprod Update.

[11] Kolibianakis EM, Bourgain C, Papanikolaou EG, Camus M, Tournaye H, Van Steirteghem AC (2005). Prolongation of follicular phase by delaying hCG administration results in a higher incidence of endometrial advancement on the day of oocyte retrieval in GnRH antagonist cycles. Hum Reprod.

[12] Haouzi D, Assou S, Mahmoud K, Tondeur S, Reme T, Hedon B (2009). Gene expression profile of human endometrial receptivity: comparison between natural and stimulated cycles for the same patients. Hum Reprod.

[13] Sunkara SK, La Marca A, Seed PT, Khalaf Y (2015). Increased risk of preterm birth and low birthweight with very high number of oocytes following IVF: an analysis of 65 868 singleton live birth outcomes. Hum Reprod.

[14] Pelinck MJ, Keizer MH, Hoek A, Simons AH, Schelling K, Middelburg K (2010). Perinatal outcome in singletons after modified natural cycle IVF and standard IVF with ovarian stimulation. Eur J Obstet Gynecol Reprod Biol.

[15] Engmann L, DiLuigi A, Schmidt D, Nulsen J, Maier D, Benadiva C (2008). The use of gonadotropin-releasing hormone (GnRH) agonist to induce oocyte maturation after cotreatment with GnRH antagonist in high-risk patients undergoing in vitro fertilization prevents the risk of ovarian hyperstimulation syndrome: a prospective randomized controlled study. Fertil Steril.

[16] Humaidan P, Polyzos NP, Alsbjerg B, Erb K, Mikkelsen AL, Elbaek HO (2013). GnRHa trigger and individualized luteal phase hCG support according to ovarian response to stimulation: two prospective randomized controlled multicentre studies in IVF patients. Hum Reprod.

[17] Tavaniotou A, Albano C, Smitz J, Devroey P (2002). Effect of clomiphene citrate on follicular and luteal phase luteinizing hormone concentrations in in vitro fertilization cycles stimulated with gonadotropins and gonadotropin-releasing hormone antagonist. Fertil Steril.

[18] Derksen L, Tournaye H, Stoop D, Van Vaerenbergh I, Bourgain C, Polyzos NP (2014). Impact of clomiphene citrate during ovarian stimulation on the luteal phase after GnRH agonist trigger. Reprod Biomed Online.

